# (4*S*)-3-Methyl-5,6,7,8-tetra­hydro-4*H*-spiro­[[1,2]oxazolo[5,4-*b*]quinoline-4,3′-indole]-2′,5-dione

**DOI:** 10.1107/S1600536814000130

**Published:** 2014-01-18

**Authors:** E. Govindan, P. S. Yuvaraj, B. S. R. Reddy, S. Bangaru Sudarsan Alwar, A. SubbiahPandi

**Affiliations:** a Department of Physics, Presidency College (Autonomous), Chennai 600 005, India; b Industrial Chemistry Laboratory, Central Leather Research Institute, Adyar, Chennai 600 020, India; c Department of Chemistry, D. G. Vaishnav College (Autonomous), Arumbakkam, Chennai 600 106, India

## Abstract

In the title compound, C_18_H_15_N_3_O_3_, the dihedral angle between the mean planes of the quinoline and indole ring systems [r.m.s. deviations = 0.189 (2) and 0.027 (2) Å, respectively] is 88.65 (5)°. The cyclo­hexene ring of the quinoline ring system adopts an envelope conformation with the central –CH_2_– C atom as the flap. In the crystal, mol­ecules are linked by two pairs of N—H⋯O hydrogen bonds, forming inversion dimers, and enclosing *R*_2_^2^(14) ring motifs. This arrangement results in the formation of chains propagating along [100].

## Related literature   

For general background to indole, quinoline and pyrrolidine derivatives, see: Padwa *et al.* (1999 ▶). For puckering parameters, see: Cremer & Pople *et al.* (1975 ▶). For asymmetry parameters, see: Nardelli (1983 ▶). For hydrogen-bond motifs, see: Bernstein *et al.* (1995 ▶).
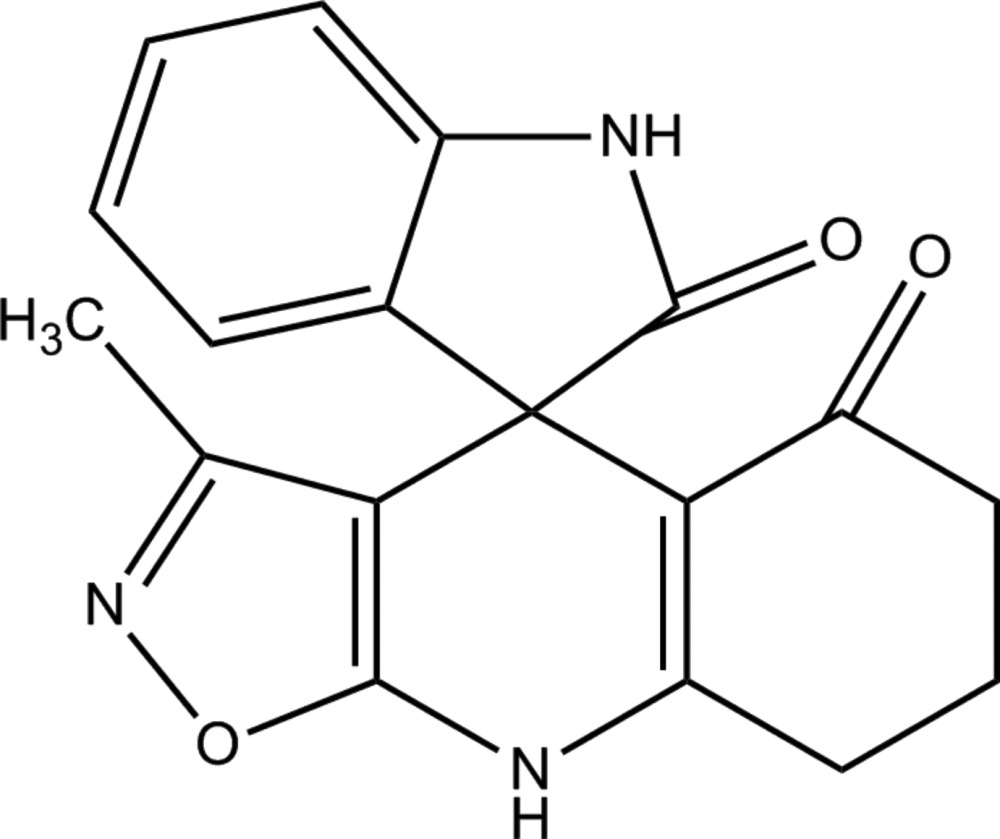


## Experimental   

### 

#### Crystal data  

C_18_H_15_N_3_O_3_
*M* *_r_* = 321.33
Monoclinic, 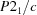

*a* = 10.9160 (3) Å
*b* = 11.9027 (3) Å
*c* = 12.4848 (4) Å
β = 111.602 (1)°
*V* = 1508.21 (7) Å^3^
*Z* = 4
Mo *K*α radiation
μ = 0.10 mm^−1^
*T* = 293 K
0.21 × 0.19 × 0.18 mm

#### Data collection  

Bruker SMART APEXII CCD diffractometer
Absorption correction: multi-scan (*SADABS*; Bruker, 2008 ▶) *T*_min_ = 0.979, *T*_max_ = 0.982
14019 measured reflections
3772 independent reflections
3088 reflections with *I* > 2σ(*I*)
*R*_int_ = 0.020

#### Refinement  

*R*[*F* ^2^ > 2σ(*F* ^2^)] = 0.047
*wR*(*F* ^2^) = 0.136
*S* = 1.04
3772 reflections
218 parameters
H-atom parameters constrained
Δρ_max_ = 0.73 e Å^−3^
Δρ_min_ = −0.35 e Å^−3^

### 

Data collection: *APEX2* (Bruker, 2008 ▶); cell refinement: *SAINT* (Bruker, 2008 ▶); data reduction: *SAINT*; program(s) used to solve structure: *SHELXS97* (Sheldrick, 2008 ▶); program(s) used to refine structure: *SHELXL97* (Sheldrick, 2008 ▶); molecular graphics: *PLATON* (Spek, 2009 ▶); software used to prepare material for publication: *SHELXL97* and *PLATON* (Spek, 2009 ▶).

## Supplementary Material

Crystal structure: contains datablock(s) global, I. DOI: 10.1107/S1600536814000130/su2673sup1.cif

Structure factors: contains datablock(s) I. DOI: 10.1107/S1600536814000130/su2673Isup2.hkl

Supporting information file. DOI: 10.1107/S1600536814000130/su2673Isup3.cml

CCDC reference:

Additional supporting information:  crystallographic information; 3D view; checkCIF report

## Figures and Tables

**Table 1:** Hydrogen-bond geometry (Å, °)

*D*—H⋯*A*	*D*—H	H⋯*A*	*D*⋯*A*	*D*—H⋯*A*
N2—H2⋯O3^i^	0.86	1.97	2.7620 (16)	153
N3—H3⋯O2^ii^	0.86	2.01	2.8415 (16)	161

Symmetry codes: (i) 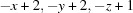; (ii) 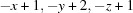.

**Fig. 1.**
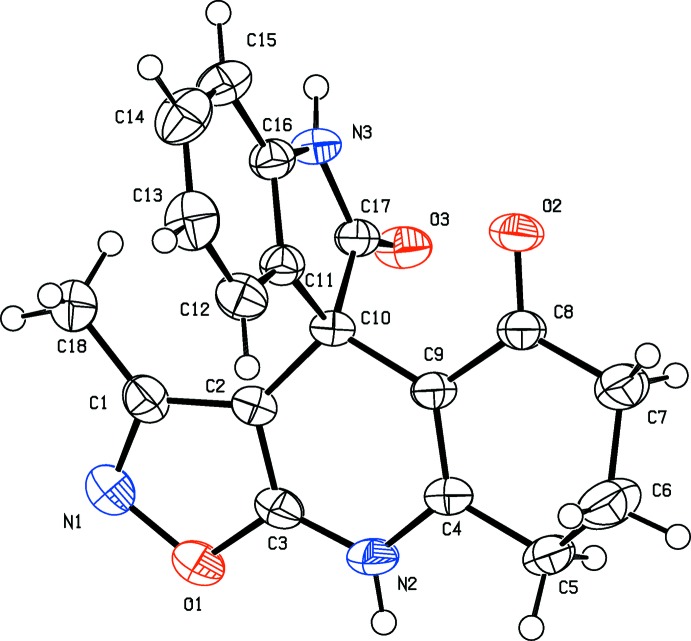
The molecular structure of the title molecule, with atom labelling. The displacement ellipsoids are drawn at the 30% probability level.

**Fig. 2.**
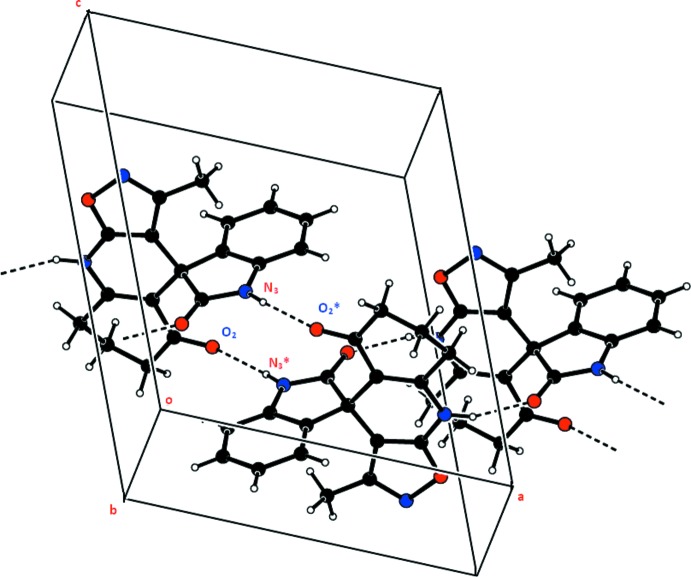
A partial view along the b-axis of the crystal packing of the title compound. It shows the two R22(14) inversion dimer formations due to the presence of two pairs of N—H···O hydrogen bonds (dashed lines; see Table 1 for details).

**Fig. 3.**
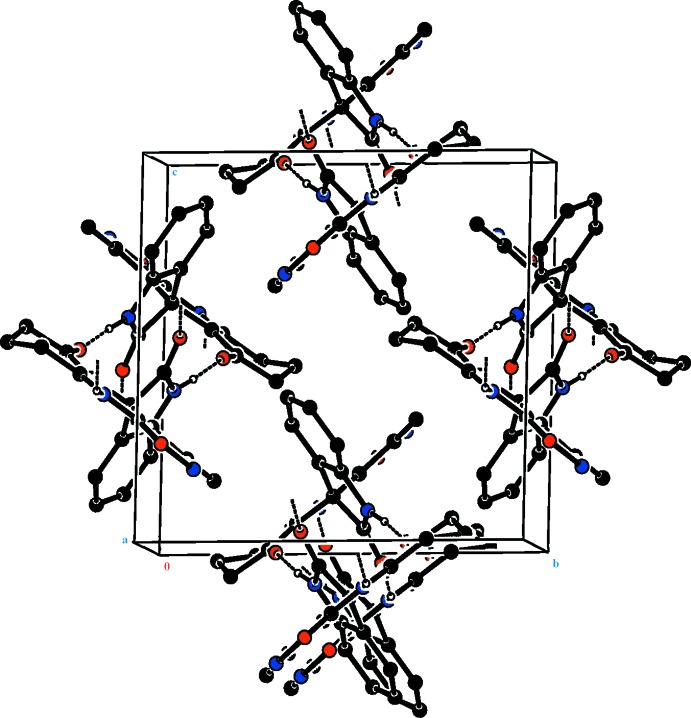
The crystal packing of the title compound viewed along the a-axis. The hydrogen bonds are shown as dashed lines (see Table 1 for details; C-bound H atoms have been omitted for clarity).

## supplementary crystallographic information

### 1. Comment

A large number of natural products contain the quinoline and indole heterocycles, and are found in numerous commercial products, including pharmaceuticals, fragrances and dyes (Padwa *et al.*, 1999). In view of the above importance we have synthesized the title compound and report herein on its crystal structure.

The molecular structure of the title molecule is shown in Fig. 1. The quinoline group and indoline ring mean planes [r.m.s = 0.189 (2) and 0.027 (2) Å, respectively] are in axial orientations with a dihedral angle of 88.65 (5)°. The indole ring adopts an almost planar conformation with a maximum deviation 0.0486 (4) Å for the spiro C atom, C10. The quinoline ring system has an envelope conformation with the puckering parameters (Cremer & Pople, 1975) and the asymmetry parameters (Nardelli,1983) are: q_2_ = 0.3087 (2) Å, φ_2_ = 209.3 (3)° and the closest pucker descriptor is an envelope on atom C6 of the cyclohexene ring. The sum of the bond angles around atoms N2 and N3 (360 °) of both the quinoline and indole rings indicates *sp*^2^ hybridization. The keto atoms O3 and O2 deviate from the attached ring system of indole and quinoline by -0.032 (1) and -0.021 (1) Å, respectively.

In the crystal, molecules are linked by two pairs of N—H···O hydrogen bonds (Table 1), forming two inversion dimers and containing two *R*^2^_2_(14) ring motifs (Bernstein *et al.*, 1995); see Fig. 2. These interactions result in the formation of chains along the a axis direction (Fig. 3 and Table 1).

### 2. Experimental

A mixture of isatin (1 mmol), cyclohexane-1,3 dione (1 mmol) and 5-Amino-3-methylisoxazole (1 mmol) in 5 ml of ethanol was heated to 353 K for 6–10 h. The reaction was monitored by TLC. When finished the reaction mixture was filtered hot and the resulting solid products were washed with ethanol, dried in air and recrystallized from ethanol, giving colourless block-like crystals.

### 3. Refinement

N and C-bound H atoms were positioned geometrically and allowed to ride on their parent atoms: N-H = 0.86 Å, C–H = 0.93, 0.97 and 0.96 Å for CH, CH_2_ and CH_3_ H atoms, respectively, with *U*_iso_(H) = 1.5*U*_eq_(C-methyl) and = 1.2*U*_eq_(N,C) for other H atoms.

### Crystal data

**Table:**

C_18_H_15_N_3_O_3_	*F*(000) = 672
*M**_r_* = 321.33	*D*_x_ = 1.415 Mg m^−^^3^
Monoclinic, *P*2_1_/*c*	Mo *K*α radiation, λ = 0.71073 Å
Hall symbol: -P 2ybc	Cell parameters from 3088 reflections
*a* = 10.9160 (3) Å	θ = 2.0–28.4°
*b* = 11.9027 (3) Å	µ = 0.10 mm^−^^1^
*c* = 12.4848 (4) Å	*T* = 293 K
β = 111.602 (1)°	Block, colourless
*V* = 1508.21 (7) Å^3^	0.21 × 0.19 × 0.18 mm
*Z* = 4	

### Data collection

**Table:**

Bruker SMART APEXII CCD diffractometer	3772 independent reflections
Radiation source: fine-focus sealed tube	3088 reflections with *I* > 2σ(*I*)
Graphite monochromator	*R*_int_ = 0.020
ω and φ scans	θ_max_ = 28.4°, θ_min_ = 2.0°
Absorption correction: multi-scan (*SADABS*; Bruker, 2008)	*h* = −14→14
*T*_min_ = 0.979, *T*_max_ = 0.982	*k* = −15→15
14019 measured reflections	*l* = −16→15

### Refinement

**Table:**

Refinement on *F*^2^	Primary atom site location: structure-invariant direct methods
Least-squares matrix: full	Secondary atom site location: difference Fourier map
*R*[*F*^2^ > 2σ(*F*^2^)] = 0.047	Hydrogen site location: inferred from neighbouring sites
*wR*(*F*^2^) = 0.136	H-atom parameters constrained
*S* = 1.04	*w* = 1/[σ^2^(*F*_o_^2^) + (0.067*P*)^2^ + 0.5984*P*] where *P* = (*F*_o_^2^ + 2*F*_c_^2^)/3
3772 reflections	(Δ/σ)_max_ < 0.001
218 parameters	Δρ_max_ = 0.73 e Å^−^^3^
0 restraints	Δρ_min_ = −0.35 e Å^−^^3^

### Special details

**Table:**

Geometry. All e.s.d.'s (except the e.s.d. in the dihedral angle between two l.s. planes) are estimated using the full covariance matrix. The cell e.s.d.'s are taken into account individually in the estimation of e.s.d.'s in distances, angles and torsion angles; correlations between e.s.d.'s in cell parameters are only used when they are defined by crystal symmetry. An approximate (isotropic) treatment of cell e.s.d.'s is used for estimating e.s.d.'s involving l.s. planes.
Refinement. Refinement of *F*^2^ against ALL reflections. The weighted *R*-factor *wR* and goodness of fit *S* are based on *F*^2^, conventional *R*-factors *R* are based on *F*, with *F* set to zero for negative *F*^2^. The threshold expression of *F*^2^ > σ(*F*^2^) is used only for calculating *R*-factors(gt) *etc*. and is not relevant to the choice of reflections for refinement. *R*-factors based on *F*^2^ are statistically about twice as large as those based on *F*, and *R*- factors based on ALL data will be even larger.

### Fractional atomic coordinates and isotropic or equivalent isotropic displacement parameters (Å^2^)

**Table:**

	*x*	*y*	*z*	*U*_iso_*/*U*_eq_	
C1	0.75216 (16)	1.11349 (13)	0.22069 (13)	0.0379 (3)	
C2	0.78149 (13)	1.02411 (12)	0.30195 (12)	0.0305 (3)	
C3	0.90595 (14)	0.99600 (13)	0.31647 (13)	0.0352 (3)	
C4	0.91557 (13)	0.84446 (12)	0.43585 (12)	0.0318 (3)	
C5	0.99809 (15)	0.74920 (14)	0.50327 (15)	0.0404 (3)	
H5A	1.0527	0.7208	0.4631	0.048*	
H5B	1.0559	0.7765	0.5780	0.048*	
C6	0.9153 (2)	0.65604 (17)	0.5196 (2)	0.0634 (6)	
H6A	0.9715	0.6024	0.5742	0.076*	
H6B	0.8730	0.6175	0.4468	0.076*	
C7	0.8122 (2)	0.69692 (18)	0.5624 (2)	0.0632 (6)	
H7A	0.8541	0.7163	0.6432	0.076*	
H7B	0.7508	0.6362	0.5565	0.076*	
C8	0.73613 (14)	0.79727 (13)	0.49828 (13)	0.0356 (3)	
C9	0.78951 (13)	0.86471 (11)	0.42928 (12)	0.0297 (3)	
C10	0.70456 (12)	0.96318 (11)	0.36267 (11)	0.0281 (3)	
C11	0.56686 (13)	0.93371 (12)	0.27954 (12)	0.0309 (3)	
C12	0.52498 (16)	0.86478 (14)	0.18423 (13)	0.0395 (3)	
H12	0.5849	0.8222	0.1644	0.047*	
C13	0.39008 (18)	0.86039 (15)	0.11791 (15)	0.0477 (4)	
H13	0.3598	0.8141	0.0535	0.057*	
C14	0.30162 (17)	0.92411 (16)	0.14721 (16)	0.0501 (4)	
H14	0.2125	0.9201	0.1017	0.060*	
C15	0.34238 (15)	0.99406 (15)	0.24296 (15)	0.0433 (4)	
H15	0.2825	1.0366	0.2628	0.052*	
C16	0.47563 (13)	0.99759 (12)	0.30727 (12)	0.0320 (3)	
C17	0.67211 (13)	1.04533 (11)	0.44623 (12)	0.0294 (3)	
C18	0.62743 (19)	1.17717 (16)	0.16867 (16)	0.0501 (4)	
H18A	0.6439	1.2452	0.1349	0.075*	
H18B	0.5932	1.1953	0.2272	0.075*	
H18C	0.5644	1.1322	0.1102	0.075*	
N1	0.85270 (15)	1.13783 (13)	0.19114 (13)	0.0492 (4)	
N2	0.97711 (12)	0.91031 (12)	0.38120 (12)	0.0402 (3)	
H2	1.0571	0.8979	0.3876	0.048*	
N3	0.54091 (11)	1.06336 (10)	0.40459 (10)	0.0328 (3)	
H3	0.5020	1.1098	0.4343	0.039*	
O1	0.95520 (11)	1.05973 (11)	0.25399 (11)	0.0477 (3)	
O2	0.63136 (11)	0.82301 (10)	0.50785 (11)	0.0443 (3)	
O3	0.75076 (10)	1.08902 (10)	0.53253 (9)	0.0400 (3)	

### Atomic displacement parameters (Å^2^)

**Table:**

	*U*^11^	*U*^22^	*U*^33^	*U*^12^	*U*^13^	*U*^23^
C1	0.0425 (8)	0.0408 (8)	0.0340 (7)	−0.0003 (6)	0.0181 (6)	0.0004 (6)
C2	0.0302 (6)	0.0326 (7)	0.0325 (7)	−0.0004 (5)	0.0159 (5)	−0.0017 (5)
C3	0.0315 (7)	0.0414 (8)	0.0382 (7)	−0.0035 (6)	0.0193 (6)	−0.0007 (6)
C4	0.0270 (6)	0.0347 (7)	0.0361 (7)	0.0020 (5)	0.0144 (5)	−0.0035 (5)
C5	0.0308 (7)	0.0422 (8)	0.0499 (8)	0.0099 (6)	0.0168 (6)	0.0033 (7)
C6	0.0460 (10)	0.0454 (10)	0.1040 (17)	0.0139 (8)	0.0338 (11)	0.0199 (10)
C7	0.0557 (11)	0.0594 (12)	0.0914 (15)	0.0243 (9)	0.0471 (11)	0.0374 (11)
C8	0.0330 (7)	0.0350 (7)	0.0446 (8)	0.0048 (6)	0.0210 (6)	0.0029 (6)
C9	0.0262 (6)	0.0303 (7)	0.0354 (7)	0.0025 (5)	0.0147 (5)	−0.0007 (5)
C10	0.0242 (6)	0.0308 (7)	0.0325 (6)	0.0012 (5)	0.0142 (5)	−0.0018 (5)
C11	0.0275 (6)	0.0319 (7)	0.0344 (7)	0.0000 (5)	0.0129 (5)	0.0006 (5)
C12	0.0408 (8)	0.0411 (8)	0.0394 (8)	−0.0027 (6)	0.0180 (6)	−0.0054 (6)
C13	0.0473 (9)	0.0492 (10)	0.0402 (8)	−0.0090 (7)	0.0084 (7)	−0.0073 (7)
C14	0.0316 (8)	0.0535 (10)	0.0532 (10)	−0.0037 (7)	0.0016 (7)	−0.0006 (8)
C15	0.0274 (7)	0.0456 (9)	0.0536 (9)	0.0042 (6)	0.0110 (7)	0.0004 (7)
C16	0.0267 (6)	0.0332 (7)	0.0368 (7)	0.0010 (5)	0.0124 (5)	0.0012 (5)
C17	0.0265 (6)	0.0306 (7)	0.0341 (7)	0.0019 (5)	0.0147 (5)	−0.0004 (5)
C18	0.0540 (10)	0.0531 (10)	0.0483 (9)	0.0138 (8)	0.0247 (8)	0.0146 (8)
N1	0.0489 (8)	0.0562 (9)	0.0500 (8)	0.0036 (7)	0.0271 (7)	0.0130 (7)
N2	0.0259 (6)	0.0485 (8)	0.0521 (8)	0.0051 (5)	0.0212 (6)	0.0070 (6)
N3	0.0265 (6)	0.0345 (6)	0.0402 (6)	0.0047 (4)	0.0155 (5)	−0.0044 (5)
O1	0.0399 (6)	0.0584 (7)	0.0549 (7)	0.0002 (5)	0.0293 (5)	0.0115 (6)
O2	0.0385 (6)	0.0459 (6)	0.0609 (7)	0.0082 (5)	0.0328 (6)	0.0084 (5)
O3	0.0289 (5)	0.0498 (6)	0.0411 (6)	−0.0005 (4)	0.0125 (4)	−0.0127 (5)

### Geometric parameters (Å, º)

**Table:**

C1—N1	1.312 (2)	C10—C11	1.5201 (18)
C1—C2	1.423 (2)	C10—C17	1.5628 (18)
C1—C18	1.483 (2)	C11—C12	1.377 (2)
C2—C3	1.3451 (19)	C11—C16	1.3941 (19)
C2—C10	1.5082 (18)	C12—C13	1.400 (2)
C3—O1	1.3348 (17)	C12—H12	0.9300
C3—N2	1.355 (2)	C13—C14	1.379 (3)
C4—N2	1.3668 (19)	C13—H13	0.9300
C4—C9	1.3695 (18)	C14—C15	1.389 (3)
C4—C5	1.499 (2)	C14—H14	0.9300
C5—C6	1.491 (3)	C15—C16	1.379 (2)
C5—H5A	0.9700	C15—H15	0.9300
C5—H5B	0.9700	C16—N3	1.3998 (19)
C6—C7	1.494 (3)	C17—O3	1.2195 (17)
C6—H6A	0.9700	C17—N3	1.3487 (17)
C6—H6B	0.9700	C18—H18A	0.9600
C7—C8	1.506 (2)	C18—H18B	0.9600
C7—H7A	0.9700	C18—H18C	0.9600
C7—H7B	0.9700	N1—O1	1.4440 (19)
C8—O2	1.2315 (17)	N2—H2	0.8600
C8—C9	1.448 (2)	N3—H3	0.8600
C9—C10	1.5354 (19)		
			
N1—C1—C2	111.93 (14)	C2—C10—C17	109.86 (11)
N1—C1—C18	119.47 (14)	C11—C10—C17	100.95 (10)
C2—C1—C18	128.59 (14)	C9—C10—C17	110.86 (11)
C3—C2—C1	103.48 (13)	C12—C11—C16	119.94 (13)
C3—C2—C10	122.29 (13)	C12—C11—C10	131.11 (13)
C1—C2—C10	134.21 (13)	C16—C11—C10	108.77 (12)
O1—C3—C2	112.58 (14)	C11—C12—C13	118.39 (15)
O1—C3—N2	120.71 (13)	C11—C12—H12	120.8
C2—C3—N2	126.63 (13)	C13—C12—H12	120.8
N2—C4—C9	122.47 (13)	C14—C13—C12	120.63 (16)
N2—C4—C5	114.19 (12)	C14—C13—H13	119.7
C9—C4—C5	123.34 (13)	C12—C13—H13	119.7
C6—C5—C4	111.73 (13)	C13—C14—C15	121.62 (15)
C6—C5—H5A	109.3	C13—C14—H14	119.2
C4—C5—H5A	109.3	C15—C14—H14	119.2
C6—C5—H5B	109.3	C16—C15—C14	117.02 (15)
C4—C5—H5B	109.3	C16—C15—H15	121.5
H5A—C5—H5B	107.9	C14—C15—H15	121.5
C5—C6—C7	112.39 (17)	C15—C16—C11	122.38 (14)
C5—C6—H6A	109.1	C15—C16—N3	127.81 (13)
C7—C6—H6A	109.1	C11—C16—N3	109.79 (12)
C5—C6—H6B	109.1	O3—C17—N3	125.10 (13)
C7—C6—H6B	109.1	O3—C17—C10	126.70 (12)
H6A—C6—H6B	107.9	N3—C17—C10	108.16 (11)
C6—C7—C8	114.14 (16)	C1—C18—H18A	109.5
C6—C7—H7A	108.7	C1—C18—H18B	109.5
C8—C7—H7A	108.7	H18A—C18—H18B	109.5
C6—C7—H7B	108.7	C1—C18—H18C	109.5
C8—C7—H7B	108.7	H18A—C18—H18C	109.5
H7A—C7—H7B	107.6	H18B—C18—H18C	109.5
O2—C8—C9	120.75 (13)	C1—N1—O1	105.43 (12)
O2—C8—C7	119.74 (14)	C3—N2—C4	116.76 (12)
C9—C8—C7	119.47 (13)	C3—N2—H2	121.6
C4—C9—C8	118.85 (13)	C4—N2—H2	121.6
C4—C9—C10	124.05 (12)	C17—N3—C16	112.00 (11)
C8—C9—C10	116.77 (11)	C17—N3—H3	124.0
C2—C10—C11	111.19 (11)	C16—N3—H3	124.0
C2—C10—C9	107.60 (10)	C3—O1—N1	106.57 (11)
C11—C10—C9	116.23 (11)		
			
N1—C1—C2—C3	−1.17 (18)	C9—C10—C11—C12	−60.3 (2)
C18—C1—C2—C3	178.32 (17)	C17—C10—C11—C12	179.74 (15)
N1—C1—C2—C10	−179.41 (15)	C2—C10—C11—C16	−111.75 (13)
C18—C1—C2—C10	0.1 (3)	C9—C10—C11—C16	124.72 (13)
C1—C2—C3—O1	0.85 (17)	C17—C10—C11—C16	4.74 (14)
C10—C2—C3—O1	179.36 (12)	C16—C11—C12—C13	−0.5 (2)
C1—C2—C3—N2	−176.02 (15)	C10—C11—C12—C13	−175.05 (15)
C10—C2—C3—N2	2.5 (2)	C11—C12—C13—C14	0.3 (3)
N2—C4—C5—C6	−156.45 (16)	C12—C13—C14—C15	−0.3 (3)
C9—C4—C5—C6	23.7 (2)	C13—C14—C15—C16	0.4 (3)
C4—C5—C6—C7	−49.1 (2)	C14—C15—C16—C11	−0.6 (2)
C5—C6—C7—C8	47.0 (3)	C14—C15—C16—N3	177.92 (15)
C6—C7—C8—O2	164.00 (19)	C12—C11—C16—C15	0.7 (2)
C6—C7—C8—C9	−18.2 (3)	C10—C11—C16—C15	176.31 (14)
N2—C4—C9—C8	−174.34 (13)	C12—C11—C16—N3	−178.08 (13)
C5—C4—C9—C8	5.5 (2)	C10—C11—C16—N3	−2.43 (16)
N2—C4—C9—C10	−1.1 (2)	C2—C10—C17—O3	−66.12 (18)
C5—C4—C9—C10	178.73 (13)	C11—C10—C17—O3	176.41 (14)
O2—C8—C9—C4	169.38 (15)	C9—C10—C17—O3	52.67 (19)
C7—C8—C9—C4	−8.4 (2)	C2—C10—C17—N3	111.85 (13)
O2—C8—C9—C10	−4.4 (2)	C11—C10—C17—N3	−5.62 (14)
C7—C8—C9—C10	177.81 (16)	C9—C10—C17—N3	−129.36 (12)
C3—C2—C10—C11	−132.98 (14)	C2—C1—N1—O1	1.00 (18)
C1—C2—C10—C11	45.0 (2)	C18—C1—N1—O1	−178.54 (15)
C3—C2—C10—C9	−4.65 (18)	O1—C3—N2—C4	−175.45 (13)
C1—C2—C10—C9	173.33 (15)	C2—C3—N2—C4	1.2 (2)
C3—C2—C10—C17	116.13 (15)	C9—C4—N2—C3	−1.9 (2)
C1—C2—C10—C17	−65.9 (2)	C5—C4—N2—C3	178.32 (14)
C4—C9—C10—C2	4.05 (18)	O3—C17—N3—C16	−177.34 (14)
C8—C9—C10—C2	177.46 (12)	C10—C17—N3—C16	4.65 (16)
C4—C9—C10—C11	129.43 (14)	C15—C16—N3—C17	179.84 (15)
C8—C9—C10—C11	−57.17 (16)	C11—C16—N3—C17	−1.51 (17)
C4—C9—C10—C17	−116.09 (15)	C2—C3—O1—N1	−0.29 (17)
C8—C9—C10—C17	57.31 (16)	N2—C3—O1—N1	176.78 (14)
C2—C10—C11—C12	63.2 (2)	C1—N1—O1—C3	−0.45 (17)

### Hydrogen-bond geometry (Å, º)

**Table:**

*D*—H···*A*	*D*—H	H···*A*	*D*···*A*	*D*—H···*A*
N2—H2···O3^i^	0.86	1.97	2.7620 (16)	153
N3—H3···O2^ii^	0.86	2.01	2.8415 (16)	161

Symmetry codes: (i) −*x*+2, −*y*+2, −*z*+1; (ii) −*x*+1, −*y*+2, −*z*+1.
